# The Antioxidant Activities of *Betula etnensis* Rafin. Ethanolic Extract Exert Protective and Anti-diabetic Effects on Streptozotocin-Induced Diabetes in Rats

**DOI:** 10.3390/antiox9090847

**Published:** 2020-09-10

**Authors:** Giuseppe Antonio Malfa, Barbara Tomasello, Rosaria Acquaviva, Alfonsina La Mantia, Francesco Pappalardo, Monica Ragusa, Marcella Renis, Claudia Di Giacomo

**Affiliations:** 1 Department of Drug Science, Section of Biochemistry, University of Catania, Viale A. Doria 6, 95125 Catania, Italy; btomase@unict.it (B.T.); alfy.lamantia@gmail.com (A.L.M.); francesco.pap82@gmail.com (F.P.); renis@unict.it (M.R.); cdigiaco@unict.it (C.D.G.); 2 Department of Experimental and Clinical Medicine, University Magna Graecia of Catanzaro, 88100 Catanzaro, Italy; m.ragusa@unicz.it

**Keywords:** terpenoids, polyphenols, flavonoids, betulinic acid, insulin, oxidative stress, HO-1 (heme oxygenase-1), γ-GCS (γ-glutamyl-cysteine-synthetase)

## Abstract

Pathophysiological mechanisms correlating diabetes mellitus with associated complications are still not completely clear, even though oxidative stress seems to play a pivotal role. Literature data suggest that cell damages induced by hyperglycemia, although multifactorial, have a common pathway in oxidative/nitrosative stress. The present study evaluated the effects of *Betula etnensis* Raf. bark extract, a plant belonging to the Betulaceae family endemic to Sicily, on oxidative stress and in preventing and/or retarding diabetes-associated complications in streptozotocin diabetic rats treated with the extract at dose of 0.5 g/kg body weight per day for 28 consecutive days. The extract administration significant decreased food and water intake, fasting blood glucose, weight loss and polyuria, compared with untreated diabetic animals. Furthermore, oxidative stress markers particularly, lipid hydroperoxides (LOOH) and nitrite/nitrate levels, non-proteic thiol groups (RSH), γ-glutamyl-cysteine-synthetase (γ-GCS) activities and expression, heme oxygenase-1 (HO-1), endothelial and inducible nitric oxide synthases (i-NOS e-NOS) expression, significantly changed by streptozocin treatment, were markedly restored both in plasma and tissues together with nuclear sirtuins activity (Sirt1). Results suggested that *B. etnensis* bark alcoholic extract is able to counteract oxidative stress and to ameliorate some general parameters related to diabetes.

## 1. Introduction

The WHO defines diabetes as a multiple metabolic disorder, characterized by chronic hyperglycemia with impaired metabolism of carbohydrates, fats and proteins, resulting from defects in insulin secretion, insulin action or both [1]. Insulin deficiency produces Type 1 Diabetes Mellitus (T1DM), while insulin resistance produces Type 2 Diabetes Mellitus (T2DM). Common effector mechanisms can cause this deficit: immunological stimuli in type 1 diabetes and metabolic and/or inflammatory factors in the type 2 converge on common pathways of signal transduction leading to functional alterations and cell destruction. In both cases, oxidative stress may play a key role in determining the functional deficits that lead to the progressive loss of beta cells [2]. To date, however, there is debate as to whether Reactive Oxygen Species (ROS) formation is always a primary cause or if it is sometimes actually a result of tissue damage. In fact, the onset of insulin resistance and dysfunction of pancreatic beta cells appear to be due to increased oxidative stress with a concomitant reduction of antioxidant systems. However, oxidative stress may play a relevant role both in development of some complications of diabetes (such as vascular complications, nephropathy, diabetic liver injury [3]), and may also represent a causative pathogenic factor of insulin resistance and/or pancreatic beta cell destruction [4]. In recent years, the overall incidence of diabetes is increasing [5,6], also due to incorrect lifestyle and eating habits. A proper diet with a support of herbal preparations, in fact, may represent a source of substances with preventive activities against this pathology. Ethno-pharmacology-based studies have suggested that medicinal plants may have beneficial effects on diabetes and its complications [6,7,8,9,10,11,12,13] and it is well known that vitamins and thousands of substances of different chemical nature such as polyphenols, flavonoids and terpenes, contained in plants, are capable of increasing the antioxidant defenses [14]. Several studies reported antidiabetic activity of phyto-bioactive compounds [15], but their potential beneficial effects on human health might be not limited to their antioxidant action. Many Betula species contain flavonoids, tannins, saponins, sterols and pentacyclic triterpenoids such as betulinic acid and betulin, which might have multiple biological activities, including effects on the absorption and uptake of glucose, insulin secretion and some diabetic complications [16]. *Betula. etnensis* Raf., also known as Birch of Etna, is a deciduous tree belonging to Betulaceae family. It is a legacy of the last glaciation in Sicily [17] and it grows only on the Etna volcano, at an altitude between 1000 and 2000 m. The bark is cream-colored and rich, in particular the young branches, which consists of numerous peltate resinous glands [18]. Previous studies have indicated that *B. etnensis* extracts contain considerable quantities of polyphenols, show radical scavenging activity [19] and exhibit a significant ability to differently modulate oxidative stress in an in vitro model of colon cancer [20]. The purpose of this study was to perform a preliminary phytochemical characterization of an alcoholic bark extract of *B. etnensis* and to evaluate some in vivo effects in an experimental model of diabetes induced in rats by administration of streptozotocin (STZ). To this end, together with general parameters (such as blood glucose and insulin, amounts of water and food taken, excreted urine volume, bodyweight), we assessed some oxidative stress biochemical markers (lipid hydroperoxides (LOOH) and nitrite/nitrate levels, non-proteic thiol group (RSH) amount, activity and expression of γ-glutamyl-cysteine-synthetase (γ-GCS), endothelial and inducible nitric oxide synthases (i-NOS, e-NOS) and heme oxygenase-1 (HO-1) expression) of some organs involved in oxidative stress-induced diabetes and associated complications. In addition, in view of the roles played by Sirt1 in modulating the regulation of a variety of cellular processes associated with antioxidant and redox signaling, the same experimental in vivo model of diabetes was used to assess the effects of the extract on nuclear sirtuins activity. All these data allowed the assessment of organ damages and possible antioxidant and anti-diabetic effects of *B. etnensis* bark extract in a streptozotocin-induced diabetic rat model.

## 2. Materials and Methods

### 2.1. Drugs and Chemicals

All chemicals were purchased from Sigma-Aldrich S.r.l. (Milano, Italy), except those mentioned elsewhere.

### 2.2. Plant Material and Preparation of the Extract

*B. etnensis* bark was collected in the area around Linguaglossa (Catania, Italy) in November 2018. The specimen was obtained and authenticated by the botanist Acquaviva, one of the authors. A voucher specimen of the plant (No. 36/03) was deposited in the Department of Drug Science, University of Catania, Italy. Grinded dried bark from two/three-year-old branches of *B. etnensis* for a total amount of 2000 g, was extracted three times with 70% ethanol (1–5) at room temperature under constant agitation for 24 h. The ethanolic extract was then filtered and evaporated in vacuo, to dryness with a rotatory evaporator since to obtain a brown residue. The yield of the bark extract, compared to 100 g of dried plant material, was 8.76%. The extract was resuspended in water before being administered to experimental animals.

### 2.3. Total Phenolic Content and Total Flavonoids Content

The concentration of total phenolic compounds was determined spectrophotometrically, using the Folin-Ciocalteau total phenols procedure, as previously described [21]. The concentration of total phenolic compounds was determined by comparing the absorbance between the extract and the gallic acid standard solutions and was estimated as gallic acid equivalent and expressed in mg gallic acid equivalent/g extract. Each result represents the mean ± S.D. of three experimental determinations. The flavonoid content was measured using a colorimetric assay [21]. A standard curve of catechin was used for quantification. The total flavonoid content of the extract was expressed as mg catechin equivalent/g extract. Each result represents the mean ± S.D. of three experimental determinations.

### 2.4. HPLC/DAD Analysis

One gram of each sample was extracted with 20 mL of methanol/water (9:1) and chloridric acid 1%, solution at room temperature (25 ± 2 °C) and in darkness for 24 h. The obtained solutions were filtered in a Büchner funnel and residual methanol from the solution was removed by nitrogen automated rapid evaporation (Rapid Mini of the Crescent Scientific, Mumbai, Maharashtra India). The obtained solution was purified using a Bond Elut C18 (500 mg 6 mL) column (Agilent, Santa Clara, CA, USA) according to the manufacturer’s instructions. The solid samples obtained were dissolved in 1 mL of methanol/water solution (1:1) with a final concentration of 30 mg/mL. HPLC-DAD analyses were carried out in duplicate and performed using an Agilent 1100 Infinity (Agilent, Santa Clara, CA, USA), equipped with a diode array detector (DAD) and with a 250 × 4.6 mm i.d., 5 µm Symmetry Shield RP 18 column; the mobile phases: 100% organic solvent (MeOH) in isocratic conditions; total time 35 min. The column temperature was maintained at 25 °C. The flow was 1 mL/min and the injection volume was 10 μL. The chromatogram profiles were recorded from 190 to 500 nm and monitored at 210, 280 nm ± 2 nm. HPLC-grade solvents, methanol, water, anhydrous sodium sulfate and chloridric acid were obtained from Carlo Erba Reagenti (Milano, Italy). The reference compound (betulinic acid) was obtained from PhytoLab GmbH & Co. (Vestenbergsgreuth, Germany).

### 2.5. Antioxidant Characterization of the Extract

#### 2.5.1. SOD-like Activity

The scavenger effect of the bark extract on superoxide anion (SOD-like activity) was performed as previously reported [21], and recorded as decrease in absorbance at λ = 340 nm. Results are expressed as the percentage of inhibition of NADH oxidation (IC_50_), and SOD (80 mU) was used as reference compound. The result represents the average of three independent experiments, and is reported as mean 50% inhibitory concentration (IC_50_) ± S.D. 

#### 2.5.2. DPPH Radical Scavenging Activity Assay

The free radical-scavenging capacity was tested by the ability of the extract to bleach stable 1,1-diphenyl-2-picryl-hydrazyl radical (DPPH) compared to Trolox (30 μM) a water-soluble derivative of vitamin E as reference compound. After 10 min at room temperature the absorbance at λ = 517 nm of the DPPH reaction mixture, with the different concentrations of acetonic extract in 1 mL of ethanol was recorded [22]. The result was obtained from the average of three independent experiments, and is reported as the mean 50% inhibitory concentration (IC_50_) ± S.D.

### 2.6. Animals and Treatments

Male Wistar albino rats (60 days old, 200 + 15 g b.w.) were purchased from Charles River Laboratories, Italia, s.r.l., Calco (Lecco). All the experimental procedures reported in this study met the guidelines of the Animal Care and Use Committee of University of Catania, Catania, Italy (approval number 170). Male Wistar albino rats were fed balanced standard rodent diet and kept in temperature (20 °C) and humidity (50%) controlled rooms. Animals were randomly subdivided into four experimental groups (10 rats for each group) as follows: control (Ctr.), diabetic (Diab.), non-diabetic rats treated with *B. etnensis* extract (Extr.), diabetic rats treated with *B. etnensis* extract (Diab. + Extr.); Ctr. and Extr. groups received a single intraperitoneal (i.p.) injection of 0.1 mol/L citrate buffer; Diab. and Diab. + Extr. groups received an injection of freshly prepared streptozotocin (65 mg/kg body weight) in 0.1 mol/L citrate buffer (pH 4.5). After 72 h of streptozotocin injection, blood was extracted from the tail vein for glucose analysis performed by glucometer kit (FreeStyle Optium, Abbott, UK); rats with blood glucose higher than 250 mg/dL in the fasting state were considered diabetic. Both non-diabetic (Extract) and diabetic rats (Diab. + Extract) were treated orally for 4 weeks with the extract of *B. etnensis* at the dose of 0.5 g/kg, p.o. The dose was determined according to the literature data [23,24]. Rats were placed in individual metabolic cages; in all four groups of animals, blood glucose and insulin, body weight, volume of excreted urine, amount of water and food consumed were assessed at 8, 15, 21 and 28 days. After 28 days, animals were sacrificed with an over-dose of anesthetic and blood, liver, kidney, brain and pancreas withdrawals were performed. Tissues were immediately frozen, stored at −80 °C and used within 7 days. Blood samples were collected from the caval vein of each rat in heparinized tubes. Plasma was separated by centrifugation at 800 g for 10 min at room temperature and immediately used for evaluating LOOH, RSH and nitrite/nitrate levels. Tissues were homogenized in phosphate buffer (PBS), pH 7.4; homogenates, divided into aliquots, were used to evaluate the following experimental parameters: LOOH and RSH levels, Western Blotting analysis for γ-GCS, e-NOS and i-NOS, respectively, Sirt1 and γ-GCS activities; in addition, quantitative determination of HO-1 was also carried out in each tissue.

### 2.7. Determination of LOOH, RSH and of Nitrite/Nitrate Levels in Homogenates

The determination of LOOH was performed on 200 μL of plasma or homogenate as previously reported [25], measuring the oxidation of Fe^2+^ to Fe^3+^ in the presence of xylenol orange. LOOH oxidize Fe^2+^ to Fe^3+^ in acidic solution, and the latter, in the presence of xylenol orange, forms a complex, which absorbs λ = 560 nm. The reaction mixture contained, in a total volume of 1 mL: 200 μL of plasma or homogenate, 100 μM xylenol orange, ammonium iron sulphate 250 μM, 90% methanol, 4 mM hydroxytoluene, 25 mM H_2_SO_4_. After 30 min incubation at room temperature and centrifugation at 11,000 rpm for 5′, the absorbance at λ = 560 nm was measured spectrophotometrically. The results were expressed as nmoles of LOOH/mg of protein or as μmoles/mL of plasma, using known concentrations of H_2_O_2_ (0.2–20 μM) for calibration. RSH levels were determined in 200 μL of plasma or tissue homogenates according to a partially modified Hu’s method [26]. The test is based on the spectrophotometric measure at λ = 412 nm of the reduction of the chromophore 5,5-ditiobis-2-nitrobenzoic acid (DTNB) by thiols. The amount of RSH present in the samples was obtained using known amounts of GSH for calibration. The results were expressed as nmoles of RSH/mg protein or as μmol/mL of plasma. Protein content was quantified using the Sinergy HT (Biotek, Milano, Italy) instrument by measuring the absorbance difference at λ = 280 and λ = 260. Nitrite/nitrate levels were determined using a spectrophotometric method at λ = 540 nm; the method is based on the reaction of diazocopulation of nitrite with the Griess reagent [27]. Plasma and homogenates were filtered through a centrifugal-driven 10,000 molecular weight cut-off cellulose membrane filter (Ultrafree-MC 10,000, Millipore, Bedford, MA, USA) at 10,000 g for 1 h at room temperature to remove proteins. In total, 250 μL of ultrafiltrate were incubated for 30 min at 25 °C in phosphate buffer (50 mM, pH 7.4) with NADPH (50 μM) and nitrate reductase (60 mU); to remove the excess of NADPH, which could interfere with the Griess reagent, samples were then treated with pyruvate (5 mM) and lactic dehydrogenase (1U). After 10 min, 250 μL of Griess reagent was added to the reaction mixture. The results were expressed as nmoles of nitrite and nitrate/mg of protein; calibration was obtained using known amounts of KNO_2_/KNO_3_.

### 2.8. Western Blotting Analysis

The expression of i-NOS, e-NOS, (EnzoLife, Milan, Italy), γ-GCS (Abcam, Cambridge, UK) and β-actin (Santa Cruz, Milan, Italy) was evaluated by Western blotting. To this end, aliquots of homogenate were treated with the appropriate concentration of a mixture of Sigma-Aldrich^®^ protease inhibitors. The amount of homogenate corresponding to 30 μg of protein was recovered with loading buffer (50 mM Tris-HCl, 10% *w/v* sodium dodecylsulphate (SDS), 10% *v/v* glycerol, 10% *v/v* 2-mercaptoethanol and 0.04% of bromophenol) and boiled for 5 min. The samples were then subjected to electrophoresis on SDS-PAGE (10% acrylamide at 100 volts constant) using as “electrophoretic run buffer” 5 mM Tris base containing 50 mM glycine and SDS 2% (w/v). After the electrophoretic stroke, the gels were transferred to a nitrocellulose membrane (Biorad, Hercules, CA, USA) [28]. The membranes were blocked with Tris-buffered saline containing 0.01% Tween 20 (TBST) and 5% non-fat milk, washed briefly and incubated overnight with the specific primary antibody (a 1:1000 dilution of γ-GCS, and 1:500 dilution of anti-eNOS, iNOS). After washing with TBST, the membranes were incubated for 1 h with the specific anti-mouse antibody. The Ag-Ab complex was highlighted using a secondary antibody, conjugated to a dye absorbing at λ = 800 or 700 nm. The membranes were analyzed using the Odyssey Infrared Imaging Scanner (Li-Cor Science Tec, Lincoln, NE, USA) and quantified by densitometric analysis. The results, normalized with β-actin, were expressed as Arbitrary Units (U.A.).

### 2.9. Enzymatic Activity Assay and Enzyme-Linked Immunosorbent Assay

For the Enzymatic activity of γ-GCS aliquots of homogenate were centrifuged at 12,000× *g* for 15 min at 4 °C. The dosage was performed on a quantity of supernatant corresponding to 100 μg of proteins according to Nakajima et al. [29]. Samples were treated with 100 mM TRIS-HCl/150 mM KCl buffer containing 5 mM Na_2_ATP, 2 mM phosphoenolpyruvate, 10 mM monosodic glutamate, 20 mM MgCl_2_, 2 mM Na_2_EDTA, 0.2 mM NADH, 17 μg of pyruvate kinase and 17 μg of lactic acid dehydrogenase. After 2 min incubation at 37 °C, L-α-aminobutyrate (10 mM final concentration) was added. The change in absorbance at λ = 340 nm was monitored for 5 min and NADH oxidation was measured spectrophotometrically using εM = 6.22 M^−1^ [30]. The enzymatic activity was expressed as mU/mg prot. Detection and quantitative determination of HO-1 were carried out in tissue homogenates according to the instructions of an AbCam commercially available E.L.I.S.A. (Enzyme-Linked Immuno Sorbent Assay) kit, which provides the spectrophotometric determination at λ = 450 nm using a microplate reader; the concentration of HO-1 in the samples was calculated thanks to a standard curve obtained with known amounts of recombinant HO-1, included in the kit. In accordance with the instructions of the supplier company, 100 μL aliquots of homogenate were used for each determination. The results were expressed as ng/mg of proteins. Each sample was measured in triple and the mean ± S.D.

### 2.10. Sirt1 Activity

Sirt1 activity in nuclear fraction of tissue homogenates was measured by colorimetric Sirt1 activity assay kit (Abcam, Cambridge, UK) following the instructions of the manufacturer. Nuclear fraction was extracted by using the commercial nuclear extraction kit (Abcam, Cambridge, UK). SIRT1 activity was normalized to total protein content of each sample and expressed as absorbance (at λ = 450 nm)/min/mg prot. 

### 2.11. Statistical Analysis

The normality of the data distribution was evaluated using both Kolmogorov-Smirnov normality test and Shapiro–Wilk normality test. One-way analysis of variance (ANOVA) followed by Bonferroni’s t test was performed in order to estimate significant differences among groups. Data were reported as mean values ± S.D. and differences between groups were considered to be significant at *p* < 0.005.

## 3. Results

### 3.1. Phytochemical Contents and Antioxidant Activity 

**Figure 1:**
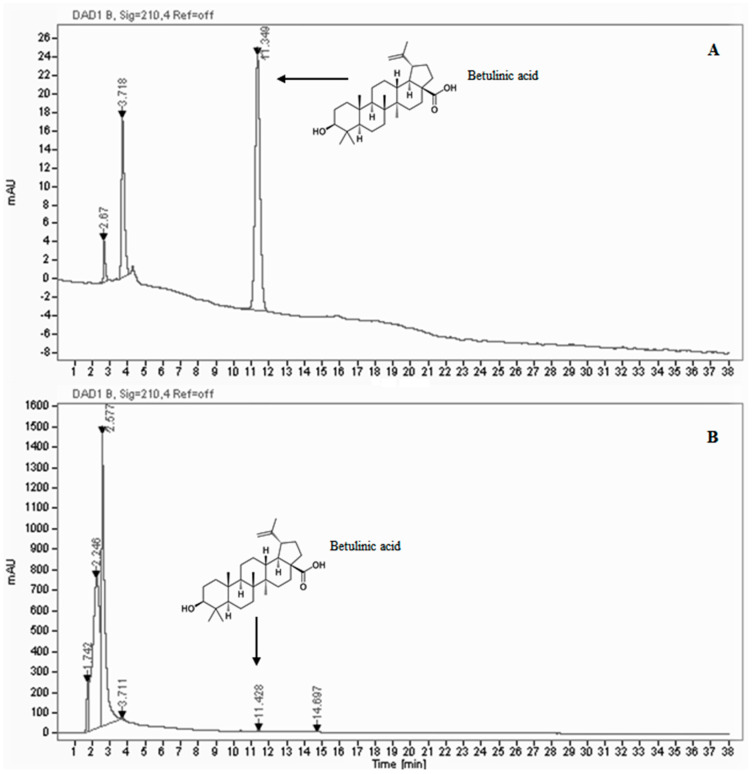
(**A**) HPLC Chromatogram of betulinic acid standard (Residence time = 11.349 min) with Chromatographic conditions: Symmetry Shield RP 18 (4.6 × 250 mm, 5 µm) column, methanol, λ = 210 nm, 0.5 mL/minutes flowrate. (**B**) HPLC Chromatogram of ethanolic extract of *B. etnensis* bark with Chromatographic conditions: Symmetry Shield RP 18 (4.6 × 250 mm, 5 µm) column, methanol, λ = 210 nm, 0.5 mL/minutes flowrate.

The total phenol and flavonoid content assays showed values of 57.30 ± 0.83 mg gallic acid equivalent (GE)/g extract and 6.11 ± 0.17 mg catechin equivalent (CE)/g extract (Table 1). In order to characterize the extract, betulinic acid was identified and quantified by HPLC/DAD analysis. The HPLC chromatograms of betulinic acid standard and *B. etnensis* bark extract are shown in Figure 1. The retention time for betulinic acid standard is 11.349 min (±0.2 min). At a retention time of 11.428 min (±0.2 min), the peak was identified as betulinic acid and by means of a calibration curve in a region of concentration between 0.01 and 0.1 mg/mL; it was quantified with a value of 0.069 mg/mL (Table 1). The extract inhibited superoxide anion formation in a dose-dependent manner. The scavenger effect showed an IC_50_ value of 0.51 ± 80 μg/mL, comparable with the IC_50_ value of 50 mU ± 0.85 of the positive control superoxide dismutase (SOD) (Table 1). The antioxidant activity, analyzed with a DPPH test, showed a concentration-dependent free radical scavenging capacity, with an IC_50_ value of 48.35 ± 1.7 μg/mL, equivalent to 15 mM ± 0.62 of Trolox (Table 1).

### 3.2. Effect of Extract Administration on Blood Glucose and Insulin, Body Weight, Volume of Excreted Urine, Amount of Water and Food Intake

As shown in Table 2, STZ induced a significant and lasting increase of glycemia; daily oral intake of Betula extract for consecutive 28 days counteracted the hyperglycemia STZ-induced, but did not modify blood glucose levels in non-diabetic rats. Coherently with hyperglycemia, despite increased food intake, a gradual and substantial weight loss was observed in untreated diabetic rats, accompanied by polydipsia and polyuria. All these effects were effectively counteracted by oral administration of the extract; in fact, compared with untreated diabetic animals, oral intake of the extract significantly reduced food and water intake, weight loss and polyuria; Table 2 also shows that the same treatment did not alter these parameters in non-diabetic, treated rats. Moreover, insulin levels were increased by the administration of the extract (Table 2).

### 3.3. Oxidative Stress in Diabetic Rat

**Figure 2:**
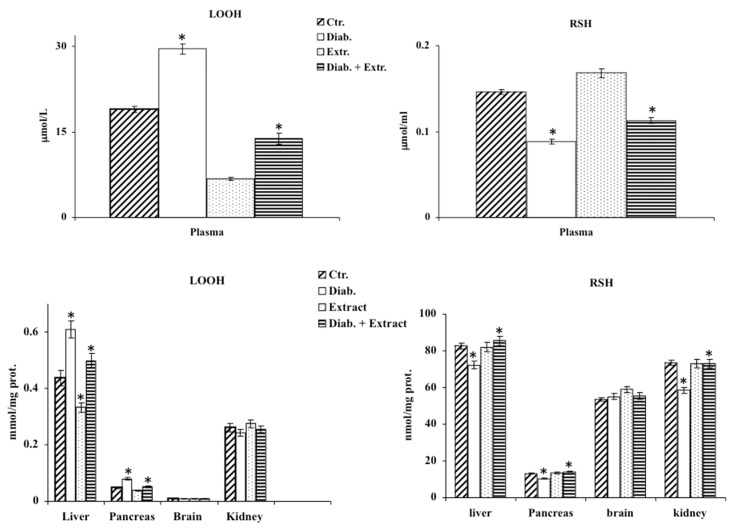
LOOH and RSH levels in plasma and in homogenates of control rats (Ctr.), diabetic rats (Diab.), non-diabetic rats treated with *B. etnensis* extract (Extr.) and diabetic rats treated with *B. etnensis* extract (Diab. + Extr.). Values represent the mean ± S.D. of four experimental determinations (10 samples/group). * *p* < 0.001 vs. Ctr and vs. Diab.

**Figure 3:**
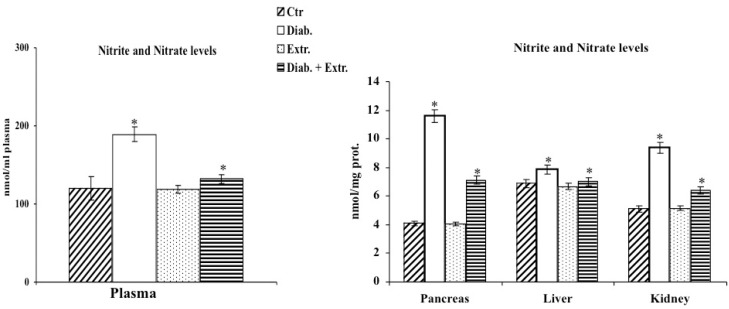
Nitrite and nitrate levels in plasma and in homogenates of control rats (Ctr.), diabetic rats (Diab.), non-diabetic rats treated with *B. etnensis* extract (Extr.) and diabetic rats treated with *B. etnensis* extract (Diab. + Extr.). Values represent the mean + S.D. of four experimental determinations (10 samples/group). * *p* < 0.001 vs. Ctr and vs. Diab.

**Figure 4:**
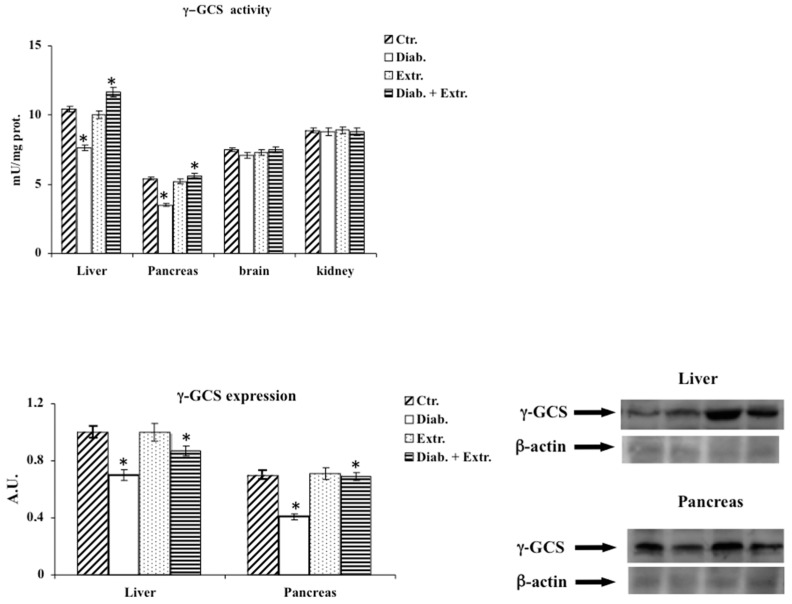
Activity and immunoblotting of γ-GCS in tissue of control rats (Ctr.), diabetic rats (Diab.), non-diabetic rats treated with *B. etnensis* extract (Extr.) and diabetic rats treated with *B. etnensis* extract (Diab. + Extr.). Values represent the mean + D.S. of four experimental determinations (10 samples/group). * *p* < 0.001 vs. Ctr and vs. Diab.

The enhanced oxidative stress induced by STZ was confirmed by the increased levels of LOOH and nitrite/nitrate in plasma, in the liver and pancreas; by the depletion of RSH in the same tissues and in the kidney; and last but not least, by the significant decrease in activity and expression of γ-GCS reported in the liver and pancreas of diabetic rats (Figure 2, Figure 3 and Figure 4). The administration of the extract mitigated the deleterious effects exerted by STZ, reducing oxidative stress by decreasing LOOH and nitrite/nitrate levels, enhancing RSH amount, activity and expression of γ-GCS in plasma, liver and pancreas (Figure 2, Figure 3 and Figure 4). The control group treated with the extract showed results comparable to the untreated control group. In this experimental model, STZ had little effect on the brain within the considered parameters, thus, we excluded it from further investigations (Figure 2, Figure 3 and Figure 4).

### 3.4. HO-1, iNOS and eNOS Protein Expression in Pancreas, Liver and Kidney

**Figure 5:**
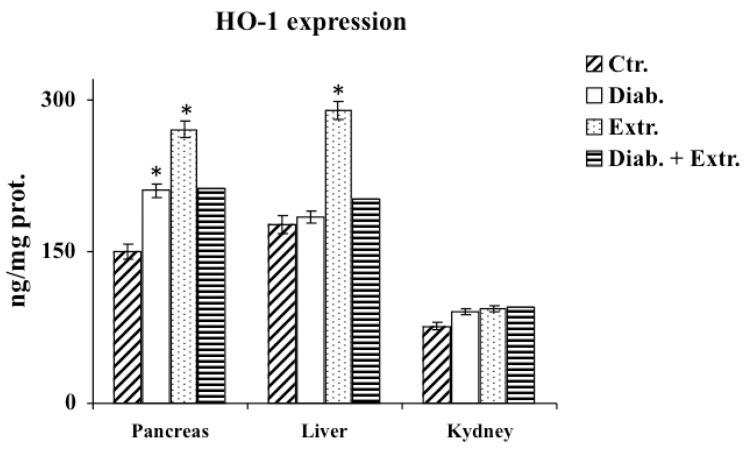
HO-1 levels in tissue of control rats (Ctr.), diabetic rats (Diab.), non-diabetic rats treated with *B. etnensis* extract (Extr.) and diabetic rats treated with *B. etnensis* extract (Diab. + Extr.). Values are the mean ± S.D. of four experimental determinations/sample (10 samples/group). * *p* < 0.001 vs. Ctr e vs. Diab.

**Figure 6:**
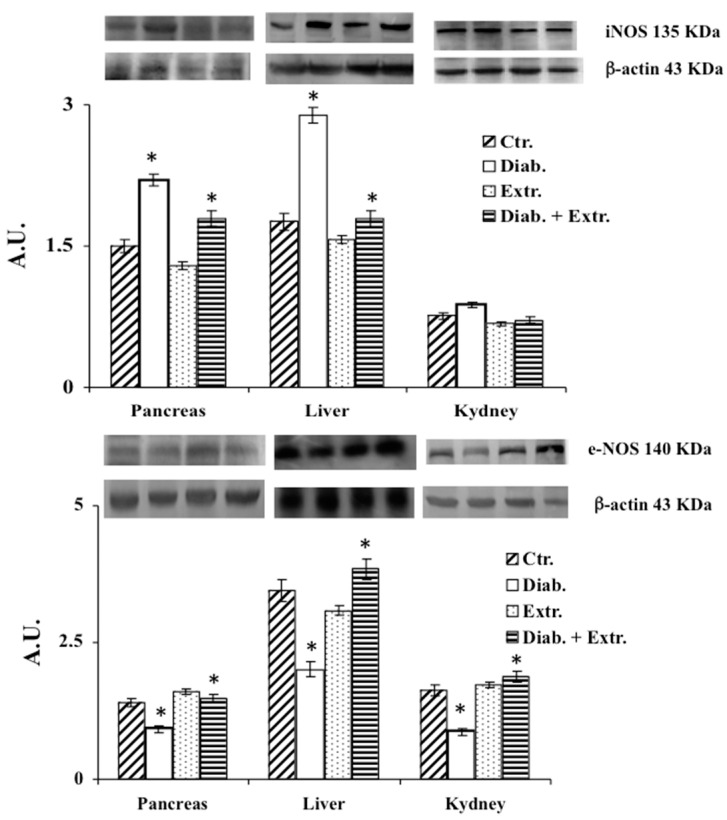
Immunoblotting of iNOS and eNOS expression in tissue of control rats (Ctr.), diabetic rats (Diab.), non-diabetic rats treated with *B. etnensis* extract (Extr.) and diabetic rats treated with *B. etnensis* extract (Diab. + Extr.). Values, expressed as Arbitrary Units, represent the mean + D.S. of four experimental determinations (10 samples/group). * *p* < 0.001 vs. Ctr and vs. Diab.

Figure 5 shows the results for HO-1 expression detected by the E.L.I.S.A. kit. STZ significantly increased the expression of this inducible enzyme in the pancreas, and increased it slightly in the kidney. The administration of the extract enhanced the expression of HO-1 in pancreas and liver in the treated control group. iNOS expression in the examined organs of diabetic rat was significantly increased (Figure 6). The extract was able to counteract the expression of the protein induced by STZ, maintaining it at a comparable level to the control group. Conversely, e-NOS protein expression levels (Figure 6) were considerably decreased in the diabetic group, in all three organs. In the diabetic treated group, e-NOS levels were maintained at the same levels of the untreated and treated control group.

### 3.5. Sirt1 Activity in Pancreas, Liver and Kidney

**Figure 7:**
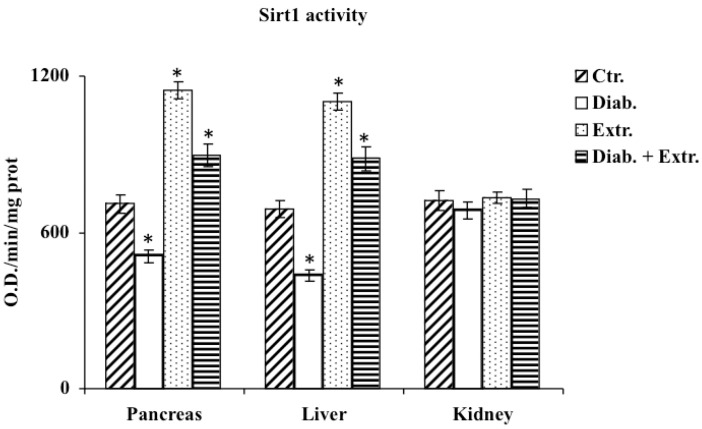
Sirt1 activity in tissue of control rats (Ctr.), diabetic rats (Diab.), non-diabetic rats treated with *B. etnensis* extract (Extr.) and diabetic rats treated with *B. etnensis* extract (Diab. + Extr.). Values are the mean ± S.D. of four experimental determinations (10 samples/group). * *p* < 0.001 vs. Ctr and vs. Diab.

Data on the activity of Sirt1 in the three organs are reported in Figure 7. STZ-induced diabetic rats showed a significant reduction in Sirt1 activity both in pancreas and liver. Instead, the administration of the extract in diabetic rat and also in control treated rat, promoted the activity of Sirt1 in pancreas and liver with values higher than in the control group. No differences were detected in the kidney among all groups.

## 4. Discussion

In diabetes, together with hyperglycemia caused by a deficiency or insensitivity to endogenous insulin, a disorder in carbohydrate metabolism is also present. Thus, the established altered state is associated with ROS overproduction that leads to a condition of oxidative stress that is involved in the pathogenesis and progression of diabetes and diabetes-associated complications [30]. In recent years, much attention has been paid to the anti-diabetic properties of many medicinal plants due to their anti-hyperglycemic, antioxidant and anti-inflammatory effects [6,7,8,9,10,11,12,13]. In this study, for the first time, we evaluated in vivo the effect of the administration of *B. etnensis* bark extract on some general diabetes-related parameters and on some key biochemical markers of oxidative stress conditions in streptozocin-induced diabetes in rats. Our results showed that daily oral intake of Betula extract counteracted the STZ-induced hyperglycemia, without modifying blood glucose levels in non-diabetic rats (Table 2) and slightly but significantly increased insulin levels in diabetic rats (Table 2) after just one week of administration. These effects restored liver homeostasis compromised by reduced peripheral glucose uptake and increased hepatic glucose production, suggesting that the extract probably enhances the release of insulin from surviving β-cells or increases the sensitivity of its receptors. STZ-induced diabetes displays all the hallmarks of functional and biochemical alterations observed in diabetes in humans such as increase in food and water intake and body weight loss due to the degradation of structural proteins [31]. The present study showed that administration of *B. etnensis* bark extract for 28 days significantly reduced food and water intakes as well as polyuria in diabetic rats, but above all improved body weight loss (Table 2), a mark of a more efficient metabolic homeostasis. In diabetes, the excessive generation of reactive oxygen species (ROS), mainly due to hyperglycemia, leads to oxidative stress in organs and tissues. Oxidative stress results from an imbalance in free radical production and endogenous antioxidant defenses. The unphysiological high levels of ROS and reactive nitrogen species (RNS) and the concomitant decrease in endogenous antioxidants generate cellular damage, thus promoting the development of diabetes associated complications [2,4]. Induction of diabetes in rats with STZ resulted in significant changes in oxidative stress markers both in plasma and tissues. Reduced glutathione is an important intracellular endogenous antioxidant and its decrease represents an oxidative stress biomarker. It is both a direct scavenger of free radicals and a cofactor for many enzymes, directly and indirectly, involved in antioxidant defense, such as glutathione peroxidase; it also participates in the thiol protection and redox regulation of protein thiol groups under oxidative stress conditions [32]. Additionally, in blood, the proportion between reduced glutathione and oxidized glutathione (GSH/GSSG) is indicative of oxidative stress conditions [33]. In our study, it was observed that the administration of STZ induced a massive reduction in RSH (whose main constituent is Glutathione, GSH) amount in plasma, liver and both at a pancreatic and renal level; conversely, no significant differences were found in the brain among the four groups and in all later determinations (Figure 2). The *B. etnensis* extract has been able to counteract the depletion of this important endogenous antioxidant; in fact, in diabetic animals treated with *B. etnensis*, the pancreatic, hepatic and renal levels of GSH were comparable to the values found in control rats (Figure 2). Lipids and in particular polyunsaturated fatty acids present in cell membranes are the main target of oxidation in supported oxidative stress status [34]. Here, reported results showed that in all three organs, hyperglycemia induced a significant increase in LOOH levels compared to control rats. The observed increase in LOOH in diabetic rats was significantly counteracted by oral treatment with the extract (Figure 2). This effect was also evident in non-diabetic treated animals and could be attributed to the antioxidant activity of the phytocomplex present in the extract (Table 1). Moreover, the significant increase in lipoperoxidation, found at the renal level, supports the hypothesis of the involvement of oxidative stress in the etiopathogenesis of diabetic nephropathy. In order to verify the hypothesis that the significant reduction of RSH levels was not only due to the depletion of endogenous antioxidant content caused by excessive ROS formation but rather due to a concomitant decrease in antioxidant capacity, in this research, the activity and expression of γ-GCS, the key enzyme in the synthesis of glutathione, were evaluated. The results obtained confirmed the hypothesis, showing that the enzymatic activity of γ-GCS in the examined organs of diabetic rats is significantly reduced compared to control animals; in addition, oral administration of birch extract was able to significantly increase the enzymatic activity compared to diabetic rats (Figure 4). A similar though less marked trend was also found concerning the expression of this enzyme. As mentioned above, ROS are not the only responsible for oxidative stress but RNS also play an important role in supporting this harmful condition [35]. Nitric oxide (NO), as well as ROS, has either beneficial or deleterious effects in cells and tissues. Indeed, NO is involved in many cellular signaling pathways [36], and it is also an oxygen-derived free radical. By its nature, NO can react with other radical species or substrates, generating RNS, responsible for nitrosative stress that influences the structure and the function of proteins [37]. The results obtained in the present study demonstrated that nitrite/nitrate plasma levels, directly correlated with ·NO production, were strongly increased, confirming its overproduction following hyperglycemia injury in diabetic untreated animals (Figure 3). Differently, in *B. etnensis*-treated diabetic rats, the daily administration of the extract induced a significant reduction of ·NO levels in all assessed tissues, particularly at renal level, where the role of ·NO should also be considered in the etiopathogenesis of diabetic nephropathy. In fact, it plays numerous roles in the kidney, as it is involved in the control of renal and glomerular hemodynamics, interfering on many stages of nephrons functions [38]. In order to identify the mechanism by which the extract exerts this effect, under the same experimental conditions, the expression of two of the enzymatic proteins responsible for the synthesis of ·NO has also been evaluated: the endothelial nitroxide synthase and the inducible isoform, both involved in diabetes-associated complications [39]. The results obtained in diabetic rats showed that the endothelial isoform is significantly reduced, while the inducible isoform displayed a significantly increased protein expression (Figure 6). Additionally, the extract was found able to counteract these reverse effects on endothelial and inducible NOS isoforms compared to controls. Furthermore, the expression pattern of iNOS reflects the results concerning nitrite and nitrate levels suggesting that the marked increase in nitrite and nitrate found in diabetic animals can be attributed to the overexpression of this isoform. Another important enzyme implicated in elevated oxidative stress conditions is HO-1 [40]. It is well known that together with its potent antioxidant effect, the increased activity of HO-1 is able to counteract the development of diabetes-associated complications [41], so that increased HO-1 expression may be considered both a marker of cell stress and a cellular defense mechanism. In the present study, HO-1 expression was evaluated in pancreas, liver and kidney. Betula extract, according to previous results, was able to induce this enzyme in all the studied organs (Figure 5), further confirming the protective effect exerted by the extract on oxidative stress diabetes-induced. Mammalian sirtuins are a class of proteins including seven NAD^+^-dependent enzymes [42]. Important functions of sirtuins include deacetylation of key proteins responsible for cellular homeostasis; as a consequence of deacetylation, in fact, activation or deactivation of enzymes involved in lipid, protein and carbohydrate metabolism occurs [43]. The roles of sirtuins in both carbohydrate and lipid metabolism is still controversial, despite many studies based on various experimental models of diabetes and insulin resistance demonstrating improved glucose tolerance, insulin secretion and sensitivity [44,45]. In fact, it has been reported that oxidative stress may induce sirtuin impairment [46], particularly Sirt1, affecting many stages of glucose metabolism in different organs and tissues [45,47]. Moreover, SIRT1 is a crucial player in the prevention of oxidative damage via a variety of mechanisms [46,48].

Determination of nuclear sirtuins activity in all four groups evidenced that the oral administration of Betula extract was able to counteract the streptozotocin-induced decrease in sirtuin activity restoring levels to those observed in control group (Figure 7). These last results suggested an involvement of Sirt1 activation in improving insulin sensitivity following extract oral administration. These last results suggested an involvement of Sirt1 activation in improving insulin sensitivity following the oral administration of the extract. Our data agrees with previous in vitro and in vivo studies that demonstrated the efficacy of different plant extracts containing polyphenolic and terpenic compounds on the glycemic profile, SIRT1 activity modulation and oxidant/antioxidant status [49,50,51].

Furthermore, the reported effects on sirtuins activity also allows us to hypothesize that the administration of the extract may counteract the deleterious effects of hyperglycemia and oxidative stress, also increasing the activity of these important modulators involved in the processes of glucose homeostasis, insulin secretion/sensitivity and redox homeostasis.

## 5. Conclusions

Taken together, the here reported results suggest that protective and anti-diabetic effects on STZ-induced diabetes in rats of *B. etnensis* bark extract are mainly due to the capacity of restoring redox homeostasis through the modulation of some regulatory enzymes that are involved in the antioxidant response, and also through the modulation of sirtuins activity strictly linked to glucose homeostasis and oxidative stress mitigation.

## Figures and Tables

**Table 1:** Betulinic acid, total phenols, flavonoids content of *B. etnensis* extract mg/g of extract; scavenger effect of extract on superoxide anion expressed as percentage of inhibition of NADH oxidation (rate of superoxide anion production was 4 nmoles/minutes), and as the capacity to bleach DPPH.

Sample	Betulinic Acid (mg/mL)	Total Phenolic Gallic Acid (mg/g)	Total Flavonoids Catechin (mg/g)	SOD-like Activity IC_50_ (μg/mL)	DPPH Test IC_50_ (μg/mL)
*B. etnensis* extract	0.069 ± 0.007	57.30 ± 0.83	6.11 ± 0.17	0.51 ± 0.80	48.35 ± 1.7
Positive control SOD				50 mU ± 0.85	
Trolox					15 mM ± 0.62

**Table 2:** Effect of *B. etnensis* (0.5 g/kg, p.o.). on insulin blood level, glycemia, food intake, body weight, water assumption and 24 h urine volume of normal and experimental rats.

Group	0	8 Days	15 Days	21 Days	28 Days
**Insulin (ng/mL)**					
Ctr.	0.97 ± 0.04	1.0 ± 0.02	0.99 ± 0.03	1.1 ± 0.04	0.99 ± 0.02
Diab.	0.87 ± 0.06	0.34 ± 0.05 *	0.38 ± 0.04 *	0.40 ± 0.06 *	0.39 ± 0.05 *
Extr.	1.0 ± 0.03	1.01 ± 0.03	1.0 ± 0.05	0.99 ± 0.03	1.02 ± 0.04
Diab. + Extr.	0.81 ± 0.02	0.60 ± 0.03 *	0.71 ± 0.03 *	0.78 ± 0.02 *	0.68 ± 0.02 *
**Glycemia (mg/dl)**					
Ctr.	80 ± 9.02	82 ± 7.8	90 ± 7.2	85 ± 6.98	90 ± 9.01
Diab.	78 ± 10.1	600 ± 25 *	575 ± 31 *	550 ± 26.1 *	500 ± 32 *
Extr.	81 ± 8.89	81 ± 8.23	82 ± 8.01	85 ± 8.2	87 ± 7.8
Diab. + Extr.	80 ± 9.03	320 ± 13.1 *	250 ± 14.3 *	280 ± 12.6 *	245 ± 10.2 *
**Food (gr/die)**					
Ctr.	10 ± 0.5	11 ± 1.65	10.5 ± 0.5	10 ± 0.5	12 ± 0.6
Diab.	10 ± 0.5	45 ± 6.75 *	50 ± 6.25 *	47 ± 5.87 *	45 ± 6.07 *
Extr.	12 ± 0.6	12 ± 1.8	11 ± 0.55	11.5 ± 0.57	11 ± 0.49
Diab. + Extr.	11 ± 0.55	32 ± 6.3 * *	28 ± 3.5 *	27 ± 3.3 *	24 ± 3.0 *
**Weight (gr)**					
Ctr.	330 ± 15	330 ± 14	350 ± 13	370 ± 14	382 ± 15
Diab.	335 ± 12	300 ± 12	280 ± 12 *	240 ± 11 *	210 ± 12 *
Extr.	320 ± 13	335 ± 12	355 ± 14	362 ± 14	378 ±15
Diab. + Extr.	335 ± 12	310 ± 10	300 ± 10	312 ± 9 * *	321 ± 8 *
**Water (ml/die)**					
Ctr.	34 ± 2	32 ± 1.5	30 ± 2	33 ± 1.5	34 ± 2
Diab.	30 ± 2	160 ± 4 *	165 ± 4 *	158 ± 5 *	167 ± 4 *
Extr.	35 ± 2	33 ± 2	35 ± 2	31 ± 1.8	32 ± 2
Diab. + Extr.	33 ± 2	89 ± 2.5 *	82 ± 3 *	75 ± 3 *	61 ± 3 *
**Urine (ml/die)**					
Ctr.	10 ± 1	11 ± 1	10.5 ± 1.2	10 ± 1	10 ± 1.3
Diab.	10 ± 1	135 ± 10 **	130 ± 9 **	138 ± 11 **	140 ± 8 **
Extr.	12 ± 1	12 ± 1	11 ± 1	11.5 ± 0.9	9.5 ± 1
Diab. + Extr.	11 ± 1	120 ± 2.5 **	78 ± 2 **	75 ± 2 **	58 ± 2 **

Control rats (Ctr.), diabetic rats (Diab.), non-diabetic rats treated with *B. etnensis* extract (Extr.) and diabetic rats treated with *B. etnensis* extract (Diab. + Extr.). Each value represents the mean ± S.D. of ten rats. * *p* < 0.001; ** *p* < 0.01 denote significant differences (Diab. vs. ctr and Diab. + Extr. vs. Diab.).
